# The Bonus Decrease

**Published:** 1921-09-10

**Authors:** 


### THE MATRONS' AND SISTERS' DEPARTMENT.

### THE BONUS DECREASE.

The gradual reduction in the cost of living has passed almost unnoticed by the careless, and we fear has brought but little ease to that large class of spenders who grasp at the opportunity of buying extras so soon as the cost of necessaries goes down. For this cause the date of September 1 has occasioned something of a shock in many quarters.

The highest point reached in the monthly official figures which record the cost of living was touched in November 1920, when it rose to 176 per cent. above pre-war prices. From that date the figures began to fall, and by May they had sunk to 126. In the Civil Service a bonus on a sliding scale rising or falling in accordance with the cost of living is accorded to all employees, and the same system is in operation under many Boards of Guardians, the Metropolitan Asylums Board, and other public bodies. From March 1921 the bonus is adjusted periodically half-yearly on the average figures for the preceding six months, a method which works out to the advantage of the workers while the rate is falling, though, should it unhappily begin once more to rise, the contrary would be the case. The first decrease in the bonus, and that a substantial one, was due on September 1, and affected a very large number of our readers. We understand that the mental hospital workers under the M.A.B. receive by agreement a fixed bonus, but it is in contemplation to alter this to one on a sliding scale.

It is very necessary that public bodies which employ female nurses should be reminded that the rate of pay which the nurses could command in 1914 was considerably below what is now recognised as equitable. Should the cost of living continue to fall until bonuses are altogether extinguished nurses' salaries would be reduced to a level which would indubitably deter worthy candidates from offering themselves for the service. This fact has been clearly recognised by one or two of the more enlightened Boards, which in announcing the reduction of bonuses have made additions to the regular salaries of their nursing staffs. Nurses under the Poor Law and other public bodies have a strong claim to sympathetic consideration when the sliding scale of bonuses is put into operation.

We fear that in far too many instances increases in pay, long overdue, have been granted under the form of a bonus when they should have been fixed additions. It is for managers of institutions carefully to consider to what extent the bonus is a grant to tide the recipient over a bad time, and to what extent a recognition that salaries have risen. In the latter case the reductions made on September 1 should be lessened by a permanent rise in the rate of pay.

The price of unskilled labour has gone up out of all proportion to the rise in the cost of nursing. The candidates, who used to be content with little besides board, lodging, uniform, and pocket-money, cannot now be lured into the institutions under double their former pay, and then for a forty-eight hours' week in place of one often extending to seventy-two hours. None can grudge them the improved conditions under which they now carry out their duties, but we plead for the women who have served many years in the lesser infirmaries, have small choice of other forms of employment, and can ill bear reductions in salaries only rendered tolerably just by the addition of the bonus.

Ever since trained nurses found their way into workhouses the nursing of the sick and aged in these institutions has been to a great extent carried out on a non-remunerative basis at the expense very often of the nurse's own health and prospects in life. It is time that more adequate pay should be awarded them than the very lowest for which they can be induced to work.

